# Probiotics Prevent Hypertension in a Murine Model of Systemic Lupus Erythematosus Induced by Toll-Like Receptor 7 Activation

**DOI:** 10.3390/nu13082669

**Published:** 2021-07-31

**Authors:** Néstor de la Visitación, Iñaki Robles-Vera, Javier Moleón-Moya, Manuel Sánchez, Rosario Jiménez, Manuel Gómez-Guzmán, Cristina González-Correa, Mónica Olivares, Marta Toral, Miguel Romero, Juan Duarte

**Affiliations:** 1 Department of Pharmacology, School of Pharmacy and Center for Biomedical Research (CIBM), University of Granada, 18071 Granada, Spain; nestorvp@correo.ugr.es (N.d.l.V.); roblesverai@ugr.es (I.R.-V.); javiermm95@ugr.es (J.M.-M.); manuelsanchezsantos@ugr.es (M.S.); rjmoleon@ugr.es (R.J.); mgguzman@ugr.es (M.G.-G.); cristinagoncor@gmail.com (C.G.-C.); miguelr@ugr.es (M.R.); 2 Instituto de Investigación Biosanitaria de Granada, 28029 Granada, Spain; 3 Centro de Investigación Biomédica en Red en Enfermedades Cardiovasculares, Instituto de Salud Carlos III, 28029 Madrid, Spain; 4 Biosearch Life, 18004 Granada, Spain; molivares@biosearchlife.com; 5 Gene Regulation in Cardiovascular Remodeling and Inflammation Group, Centro Nacional de Investigaciones Cardiovasculares (CNIC), 28029 Madrid, Spain

**Keywords:** probiotics, hypertension, endothelial dysfunction, TLR-7 activation, lupus

## Abstract

Our group tested the effects of *Lactobacillus fermentum* CECT5716 (LC40) and/or *Bifidobacterium breve* CECT7263 (BFM) in the prevention of gut dysbiosis, hypertension and endothelial dysfunction in a pharmacologically-induced model of systemic lupus erythematosus (SLE). We treated eight-week-old BALB/cByJRj mice without (Ctrl) or with the agonist of TLR-7 Imiquimod (IMQ) for 8 weeks. Concomitantly, LC40 (10^9^ CFU/mL) and BFM (10^9^ CFU/mL) were administered through oral gavage once a day. IMQ induced intestinal dysbiosis consisting of a decrease in the α-diversity measured with Chao-richness and numbers of species. LC40 and BFM did not restore these parameters. The three-dimensional principal component analysis of bacterial taxa in stool samples presented perfect clustering between Ctrl and IMQ groups. Clusters corresponding to LC40 and BFM were more akin to IMQ. BFM and LC40 were detected colonizing the gut microbiota of mice treated respectively. LC40 and BFM decreased plasma double-stranded DNA autoantibodies, and B cells in spleen, which were increased in the IMQ group. Also, LC40 and BFM treatments activated TLR9, reduced T cells activation, and Th17 polarization in mesenteric lymph nodes. Aortae from IMQ mice displayed a decreased endothelium-dependent vasodilator response to acetylcholine linked to pro-inflammatory and pro-oxidative status, which were normalized by both BFM and LC40. In conclusion, we demonstrate for the first time that the chronic treatment with LC40 or BFM prevented hypertension and endothelial dysfunction in a mouse lupus model induced by TLR-7 activation.

## 1. Introduction

Systemic lupus erythematosus (SLE) is a multisystemic chronic inflammatory autoimmune disorder, in which abnormal B lymphocytes [1] produce a high number of autoantibodies that, forming immunocomplexes, deposit along the body, causing inflammation and damage to several tissues and organs such as the kidneys, the skin or the vascular system [2,3]. This may generate a series of complications characteristic of this pathology, like renal injury or cardiovascular disease development depending on the patient [4].

Cardiovascular diseases are considered the main mortality cause among SLE patients [4]. Furthermore, hypertension is the principal risk factor in the development of renal and cardiovascular diseases [5,6]. A high number of SLE patients present a subclinical level of cardiovascular disease, which precedes the development of atherosclerosis. Among the subclinical changes present are endothelial dysfunction (with an intact muscular smooth muscle function) [7], thickening of the arterial wall and abnormalities in coronary perfusion [8]. Immune dysregulation drives to the infiltration of inflammatory cells and vascular inflammation, which intervene in the onset of endothelial dysfunction and hypertension in SLE [9,10].

The Toll-like receptors (TLRs) family is comprised of pattern-recognition receptors able to identify and be activated by a broad range of pathogen-associated molecular patterns, triggering an innate immune response as a result [11]. An increasing number of publications point to TLRs signaling dysfunction as an element involved in the onset and progression of human and genetic murine models of SLE [12,13]. TLR7 activation triggers phenotype and functional changes, distinctive of human SLE, which include high autoantibody levels and multiple organ involvement [14]. The TLR7/type 1 interferon (IFN) pathway is of a high relevance in human SLE [15]. Recently, it has been proven that TLR7 activation causes endothelial dysfunction [16] and hypertension [17], and further underscored the augmented vascular inflammation and oxidative stress, facilitated partially by IL-17, as crucial elements conducive to cardiovascular complications [17]. This inducible model of SLE, like other models of the disease [18,19,20,21], develops a shift in bacterial communities in the gut that parts from physiological conditions, commonly known as dysbiosis [22]. Interestingly, gut disbiosis was found in TLR7-dependent murine models of SLE, and bacterial translocation of *Lactobacillus reuteri* into the liver and the secondary lymph organs can drive autoimmunity, which was prevented by dietary resistant starch by suppressing the abundance and translocation of *L. reuteri* through short-chain fatty acids (SCFAs) [22]. Since immunomodulatory probiotics have been effective in the treatment of SLE in other models that also present dysbiosis [23], the objective of the present experiment was to elucidate if Lactobacillus *fermentum* CECT5716 (LC40) and/or *Bifidobacterium breve* CECT7263 (BFM) can prevent hypertension, endothelial dysfunction and intestinal dysbiosis in a murine lupus model inducible by epicutaneous administration of the TLR7 agonist imiquimod (IMQ). Taken into account that higher autoantibodies production by B cells and Th17 infiltration in vascular tissues were key events in the development of hypertension induced by TLR7 activation [17] we estudied the changes in immune cells induced by both probiotics. 

## 2. Materials and Methods

### 2.1. Probiotic Preparation and Administration

Treated groups received the probiotics LC40 or alternatively BFM (Biosearch, Granada, Spain), obtained as lyophilized powders, and suspended in water for administration through oral gavage at a dose of 10^9^ colony-forming units (CFU)/day and animal for 8 weeks. 

### 2.2. Animals and Experimental Groups

We randomly sorted 40 female 10-week-old BALB/c mice and were distributed into 4 groups: a Control group (*n* = 8), an IMQ group (*n* = 12), and two groups treated with each of the probiotics: IMQ-LC40 (*n* = 10) or IMQ-BFM (*n* = 10). All IMQ-treated groups received the TLR-7 agonist (Zyclara, Meda Salu, Madrid, Spain), at a dose of 1.25 mg per day and mouse for 8 weeks, administered 3 days a week in alternate days. 

All mice were purchased from Janvier (St Berthevin Cedex, France). Specific pathogen-free facilities at University of Granada Biological Services Unit were used to house the experimental animals, maintaining standard laboratory conditions (12 h light/dark cycle, temperature 21–22 °C, 50–70% humidity). Mice were kept in Makrolom cages (Ehret, Emmerdingen, Germany), with dust-free laboratory bedding and enrichment. As a preventive measure against mouse-to-mouse transmission of stool microorganisms, all mice were kept in separated cages. The animals received water and standard laboratory chow (SAFE A04, Augy, France) *ad libitum*. We registered body weight and food and water intake regularly.

All experimental procedures were performed in following the Guide for the Care and Use of Laboratory Animals (National Institutes of Health (NIH) publication no. 85-23, revised 1996) and accepted by the Ethics Committee of Laboratory Animals of the University of Granada (Spain) (Ref. 12/11/2017/164).

### 2.3. Blood Pressure Measurements

Systolic blood pressure (SBP) was assessed on alternative weeks, in the mornings in conscious, pre-warmed, restrained mice with tail-cuff plethysmography (digital pressure meter, LE 5001; Letica S.A., Barcelona, Spain). A minimum of seven determinations per mouse were recorded in every session and the mean was taken as the SBP level [24].

### 2.4. Plasma Determinations

At the experimental end-point, mice were euthanized using isoflurane anaesthesia. Blood samples were chilled on ice and centrifuged for 10 min at 3500 revolutions per minute (rpm) at 4 °C. The obtained plasma was frozen at −80 °C. Plasma anti-dsDNA antibody concentration levels were quantified with a Mouse Anti-dsDNA IgG ELISA Kit (Alpha Diagnostic International, San Antonio, TX, USA), and plasma lipopolysaccharide (LPS) levels were determined with the Limulus Amebocyte Lysate (LAL) chromogenic endotoxin quantitation Kit (Lonza, Valais, Switzerland), following the manufacturer’s instructions. 

### 2.5. Morphological Variables

The heart, kidneys, liver, spleen, and colon were excised, cleaned and weighed. The heart, kidney, liver and spleen weight indices were determined as a ratio of tibia length. Colon ratio was obtained from dividing its weight by its length. All samples were snap-frozen in liquid nitrogen and kept at −80 °C for storage.

### 2.6. Vascular Reactivity

Descending thoracic aortic ring-shaped segments were isolated from the animals and placed in a wire myograph (model 610M, Danish Myo Technology, Aarhus, Denmark) for isometric tension assessment as previously shown [24]. The rings were incubated in Krebs solution (in mM: 118 NaCl, 4.75 KCl, 25 NaHCO_3_, 1.2 MgSO_4_, 2 CaCl_2_, 1.2 KH_2_PO_4_ and 11 glucose) at 37 °C and 95% O_2_, 5% CO_2_ (pH ∼ 7.4). Length-tension characteristics were calculated using the software Myodaq 2.01. The segments were tensed up to 5 mN. The concentration-relaxation curves to acetylcholine (ACh; 1 nM–10 µM) were carried out in intact aortic segments pre-contracted by the thromboxane A2 analogous U46619 (10 nM) in the with or without the endothelial nitric oxide synthase (eNOS) inhibitor N^G^-nitro-L-arginine methyl ester (L-NAME, 100 μM), or the specific pan-NOX inhibitor VAS2870 (5 µM). We assayed the relaxant responses to sodium nitroprusside (SNP, 0.01 nM–10 μM) in endothelium-denuded vessels in the dark, precontracted by U46619 (10 nM). The relaxant response to ACh and SNP is shown as percentages relative to the precontraction by U46619.

### 2.7. NADPH Oxidase Activity

We analyzed NADPH oxidase activity in segments from aorta with a previously described lucigenin chemiluminescence assay [25]. Succinctly, aortic samples were incubated for 30 min at 37 °C in a physiological salt solution (pH 7.4, in mM: 119 NaCl, 20 HEPES, 4.6 KCl, 1 MgSO_4_, 0.15 Na_2_HPO_4_, 0.4 KH_2_PO_4_, 1 NaHCO_3_, 1.2 CaCl_2_ and 5.5 glucose). Then NADPH (100 mM) was added to stimulate the synthesis of superoxide anions (O_2_^−^). Lucigenin was automatically added (5 mM). Enzyme activity was assessed by determining luminescence over 200 s in a scintillation counter (Lumat LB 9507, Berthold, Germany) in 5 s intervals and then normalizing to basal values in the absence of NADPH. All samples were dried to obtain the dry weight. Enzyme activity is shown as relative luminescence units (RLU) per min per mg of tissue. 

### 2.8. In Situ Detection of Vascular Reactive Oxygen Species (ROS) Content

Unfixed segments from the thoracic aorta were cryopreserved (0.1 M PBS plus 30% sucrose for 1–2 h), included in optimum cutting temperature compound medium (Tissue-Tek; Sakura Finetechnical, Tokyo, Japan), frozen at −80 °C, and 10 μm cross-sections from the samples were cut using a cryostat (Microm International Model HM500 OM). Sections were incubated for 30 min in the described above buffer solution containing HEPES with dihydroethidium (DHE, 10 µM) and counterstained with the nuclear stain 4,6-diamidino-2-phenylindoledichlorohydrate (DAPI, 300 nM). In the following 24 h, the samples were examined with a fluorescence microscope (Leica DM IRB, Wetzlar, Germany). The microscopy preparations were imaged and ethidium and DAPI fluorescence was quantified using ImageJ (version 1.32j, NIH, http://rsb.info.nih/ij/ accessed on 10 May 2021). An estimation of ROS production was calculated from the ratio of ethidium/DAPI fluorescence [24]. In preliminary experiments, previous to the incubation with DHE, serial preparations were treated with the specific pan-NOX inhibitor VAS2870 (1 µM) for 30 min at 37 °C, indicating the specificity of this reaction.

### 2.9. Quantitative Polymerase Chain Reaction (qPCR)

For qPCR analysis, total RNA was obtained from different tissues and organs by homogenization and retrotranscribed to cDNA by a standard methodology. PCR reactions were carried out using a Techne Techgene thermocycler (Techne, Cambridge, UK). Real-time (RT)-PCR was performed to measure mRNA expression. The primer sequences utilized for amplification are listed in Table 1. Preliminary experiments were performed with different concentrations of cDNA to select non-saturating conditions for PCR amplification. Consequently, under optimal conditions, relative quantification of mRNA was determined with the SYBR Green-based RT-PCR method. Reaction efficiency was assessed utilizing a dilution series of a standard tissue sample. The ∆∆Ct method was used for quantification. The housekeeping genes ribosomal protein L13a (RPL13a), or glyceraldehyde-3-phosphate dehydrogenase (GADPH), were utilized for internal normalization.

### 2.10. Flow Cytometry

Spleens and Mesenteric lymph nodes (MLN) were excised after animal sacrifice. The samples were adequately mashed with wetted slides to reduce friction. The cells suspensions were then filtered through a cell strainer of 70 µm. Spleen samples were incubated at room temperature for 5 min with Gey’s solution for red blood cell lysis. In order to improve intracellular staining detection, 1 × 10^6^ cells were incubated with a protein transport inhibitor (BD GolgiPlug; BD Biosciences, CA, USA), and with 50 ng/mL phorbol 12-myristate 13-acetate (PMA) plus 1 μg/mL ionomycin to stimulate cytokine production. After 4.5 h, the aliquots were blocked with Fc-γ receptors to prevent nonspecific binding of our antibodies to these receptors (Miltenyi Biotec, Bergisch Gladbach, Germany), and stained with the live/dead stain as a viability dye (APC-Cy7, clone N418; Thermo Fisher Scientific, Bremen, Germany), incubating for 30 min at 4 °C. Next, cells were put into polystyrene test tubes for surface staining using anti-CD4 (PerCP-Cy, clone RM4-5; BD Biosciences), anti-CD45 (APE-eFluor 780, clone 30-F11; BD Biosciences), anti-B220 (allophycocyanin, cloneRA3-6B2; BD Biosciences, CA, USA), anti-CD25 (PE-Vio 770, clone 7D4; Miltenyi Biotec) for 20 min at 4 °C in the dark. We then followed with cell fixation, permeabilization, and intracellular staining with antibodies anti-FoxP3 (PE, cloneFJK-16s; Thermo Fisher Scientific), anti-IL-17a (PE-Cy7, clone eBio17B7; Thermo Fisher Scientific), and anti-IFN-γ (Alexa Fluor488, clone XMG1.2; Thermo Fisher Scientific) for 30 min at 4 °C in the dark. Data collection was accomplished with a flow cytometer Canto II (BD Biosciences) as previously described [19]. Figure S1 illustrates the gating strategy used.

### 2.11. DNA Extraction, 16S rRNA Gene Amplification and Bioinformatics

An extensive description of our protocol was previously published [19]. Succintly, DNA was isolated from stool samples. The V3-V4 regions of the 16S rRNA gene were amplified and barcoded. DNA samples were analyzed with a MiSeq instrument (Illumina, San Diego, CA, USA) with 23,300 paired-end read sequencing at the Unidad de Genomica (Parque Cientifico de Madrid, Madrid, Spain). Posterior analyses were carried out with 16SMetagenomics (v.1.0.1.0) from Illumina. The sequences were then clustered to an operational taxonomic unit (OTU) with USEARCH with default parameters (USERACH v.61). Quantitative Insights into Microbial Ecology (QIIME)-based alignments of representative sequences were determined (QIIME 1.94 version with comparison database GreenGenes) with PyNAST, and the Greengenes 13_8 database was employed as the template file. The Ribosome Database project algorithm was used to classify the representative sequences into specific taxa with the default database [26]. The Taxonomy Database (National Centre for Biotechnology Information) was utilized for classification and nomenclature. Bacteria were classified based on the SCFAs’ end-product, as previously described [27,28].

### 2.12. Reagents

The reagents utilized in this experiment were purchased from Sigma-Aldrich, unless otherwise stated.

### 2.13. Statistical Analysis

The ecological parameters shown in this article were determined using the QIIME pipeline (PAST ×3). Reads in each OTU were normalized to total reads in each sample. Only taxa with a percentage of reads > 0.001% were utilized. Partial Least Square (PLS) analysis was additionally used on these data to identify significative differences among groups. Linear discriminant analysis (LDA) scores above 2 were selected to be represented as significant. Taxonomy was summarized at the genus level within QIIME v.1.9.0 and uploaded to the Galaxy platform [29] to generate LEfSe/cladogram enrichment plots that considered significant enrichment at a value of *p* < 0.05, LDA score > 2.

GraphPad Prism 8 was used to process our results. Data are represented as means ± SEM of measurements. The statistical significance for the differences in SBP and vascular reactivity assays was determined by two-way analysis of variance (ANOVA) with the Tukey *post hoc* test. The rest of the variables were determined on normal distribution with Shapiro-Wilk normality test and compared using one-way ANOVA and Tukey *post hoc* test in case of normal distribution, or Mann-Whitney test or Kruskal-Wallis with Dunn’s multiple comparison test in case of abnormal distribution. *p* < 0.05 was set as significant.

## 3. Results

### 3.1. Probiotics Improve Intestinal Integrity without Preventing Gut Dysbiosis in IMQ-Treated Mice

**Figure 1:**
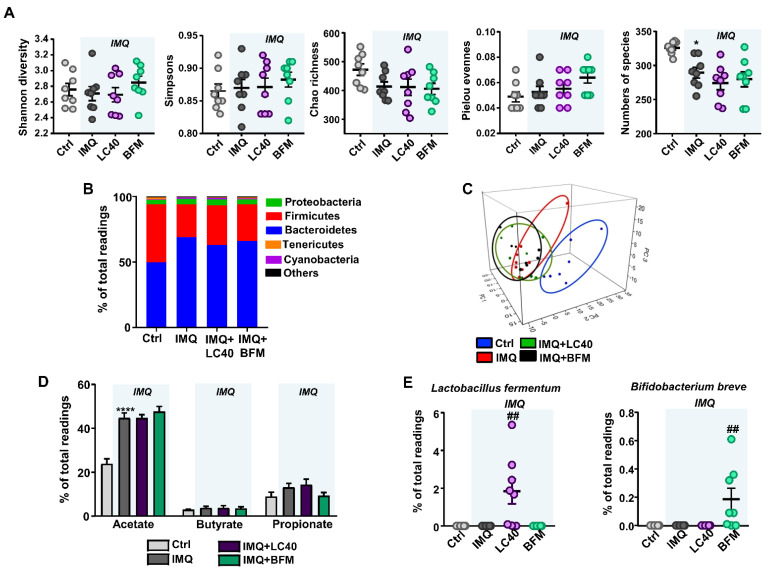
Effects of the probiotics in microecological parameters, and phyla changes of the gut microbiota from imiquimod-treated mice. (**A**) Bacterial 16S ribosomal DNA were amplified and sequenced to evaluate fecal diversity, richness, evenness and numbers of species. (**B**) Phylum breakdown of the 6 most abundant bacterial communities in the stool samples. (**C**) Principal coordinate analysis in the gut microbiota. (**D**) Proportion of short chain fatty acids (SCFAs)-producing bacteria in gut microbiota. (**E**) Bacterial species *Lactobacillus fermentum* and *Bifidobacterium breve* in the gut microbiota. Groups: control (Ctrl), Imiquimod (IMQ), IMQ treated with *Lactobacillus fermentum* CECT5716 (LC40), and IMQ-treated with *Bifidobacterium breve* CECT7263 (BFM). Results are expressed as mean ± SEM (*n* = 8). * *p* < 0.05 and **** *p* < 0.0001 compared with the Ctrl group. ^##^ *p* < 0.01 compared with the IMQ group.

To investigate whether the activation of TLR-7 pathway is linked to the presence of gut dysbiosis, we studied faecal DNA from all groups. The composition of the bacterial communities was assessed calculating relevant ecological parameters, including Shannon diversity, Simpsons, Chao richness, Pielou evenness, and the number of observed species. IMQ-treated animals display a significant reduction in α-diversity measure by the number of species, without significant changes in the other parameters. Probiotic treatments did not alter microbial richness, diversity, and evenness (Figure 1A). In a similar fashion, when we studied the phyla composition, *Firmicutes* and *Bacteroidetes* were the most abundant populations, and in lesser proportions of *Tenericutes*, *Cyanobacteria*, and *Proteobacteria* in mouse faeces. Bacteria populations from the *Firmicutes* phylum was lower in IMQ than in Ctrl. In addition to this, bacteria from *Bacteroidetes* phylum were increased in the IMQ group. Probiotics tend to decrease *Bacteroidetes* and rise *Firmicutes* but these changes were not statistically significant (Table 2, Figure 1B). The three-dimensional principal component analysis of the bacterial taxa in stool samples displayed perfect clustering among groups (Ctrl and IMQ). The clusters from both probiotic-treated groups were more similar to IMQ (Figure 1C). Figure S2 shows the bacterial taxa (class, order, family, and genus) that were altered due to TLR-7 activation, as per the LEfSe analysis. Noticeable shifts in bacterial taxa were observed at the end of the IMQ treatment, where the relative abundance of 29 taxa was increased (green) and 69 taxa were lowered (red), in comparison with the control group. All of these shifts in microbiota composition were still present in both probiotic-treated groups (not shown). In addition, the SCFAs-producing bacteria were also analysed. The levels of acetate-producing bacteria were increased in IMQ mice, but neither of the treatments restored it (Figure 1D). Despite probiotics seemed unable to prevent or reverse the changes experimented by the IMQ microbiota, LC40 and BFM were detected in the faecal samples of animals treated respectively, demonstrating that both microorganisms were able to colonize their respective hosts (Figure 1E).

**Figure 2:**
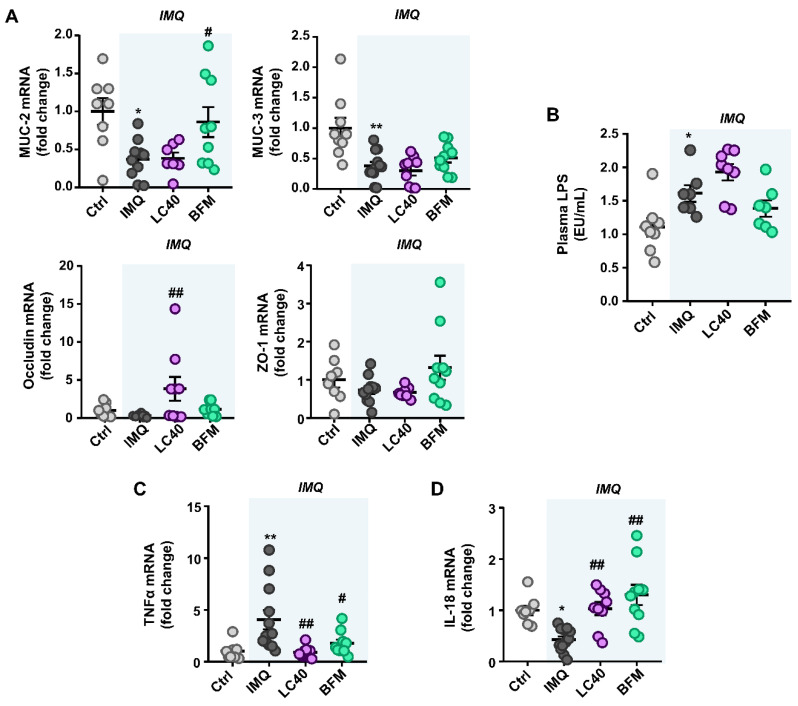
Effects of probiotic treatments on gut epithelial integrity markers in imiquimod-treated mice. (**A**) Colonic mRNA levels of mucin (MUC)-2, MUC-3, Occludin, and zonula occludens-1 (ZO-1). (**B**) Plasma endotoxin concentration (EU/mL, endotoxin units/mL). Colonic mRNA levels of cytokines TNFα (**C**) and IL-18 (**D**). Groups: control (Ctrl), Imiquimod (IMQ), IMQ treated with *Lactobacillus fermentum* CECT5716 (LC40), and IMQ-treated with *Bifidobacterium breve* CECT7263 (BFM). Results are expressed as mean ± SEM. * *p* < 0.05 and ** *p* < 0.01 compared with the Ctrl group. ^#^ *p* < 0.05 and ^##^ *p* < 0.01 compared with the IMQ group.

On the other hand, our group assessed gut barrier integrity, the colonic mRNA expression of mucin (MUC)-2 and MUC-3 was reduced in IMQ group as compared to control mice, whereas the barrier-forming junction transcripts, occludin, and zonula occludens-1 were unchanged. Interestingly, BFM treatment increased the colonic mRNA expression MUC-2 transcript, whereas LC40 significantly increased occludin levels (Figure 2A). Because of this, we determined endotoxin levels in plasma, and they were found increased in IMQ mice in comparison to the control group. However, the probiotics did not prevent endotoxemia in the IMQ group (Figure 2B). Our results suggest an increment in intestinal permeability in this mouse model of lupus, which would allow bacterial components (e.g., LPS) to enter the blood stream. Additionally, the higher mRNA levels of the colonic pro-inflammatory cytokine TNF-α in IMQ mice were decreased by LC40 and BFM administration (Figure 2C). Moreover, IL-18 colonic expression, a key cytokine in tissue repair [30] and colonic Th17 cell maturation downregulation [31], was found reduced in IMQ mice and normalized after treatment with both probiotics (Figure 2D).

### 3.2. Probiotics Attenuate Lupus Disease Activity and Modulate Immune Response

**Figure 3:**
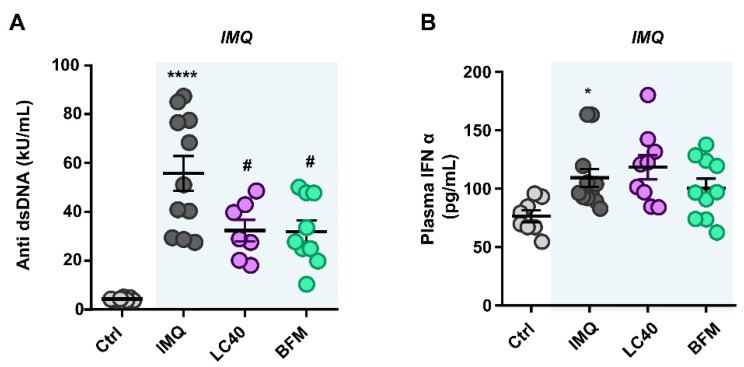
Effects of probiotic treatments on plasma markers of disease activity in imiquimod-treated mice. (**A**) Circulating double-stranded DNA autoantibodies (displayed as antibody activity index), and (**B**) interferon (IFN)-α levels in plasma from all experimental groups. Groups: control (Ctrl), Imiquimod (IMQ), IMQ treated with *Lactobacillus fermentum* CECT5716 (LC40), and IMQ-treated with *Bifidobacterium breve* CECT7263 (BFM). Results are expressed as mean ± SEM. * *p* < 0.05 and **** *p* < 0.0001 compared with the Ctrl group. ^#^ *p* < 0.05 compared with the IMQ group.

We quantified lupus disease activity through autoantibody plasma levels, and we observed a significant increment in IMQ mice in comparison with the control group (Figure 3A), as previously described [14,16,17]. Both LC40 and BMF significantly lowered anti-dsDNA levels in IMQ mice. As expected, we detected a clear splenomegaly in IMQ-treated mice (Table 3), possibly linked to autoimmune disease progression [32], but only BFM treatment was able to prevent this. SLE is a prototypical autoimmune disease characterized by a type I IFN signature [15]. In fact, higher plasma levels of IFNα were found in IMQ mice as compared to control; however, probiotic treatments were unable to change this parameter (Figure 3B).

**Figure 4:**
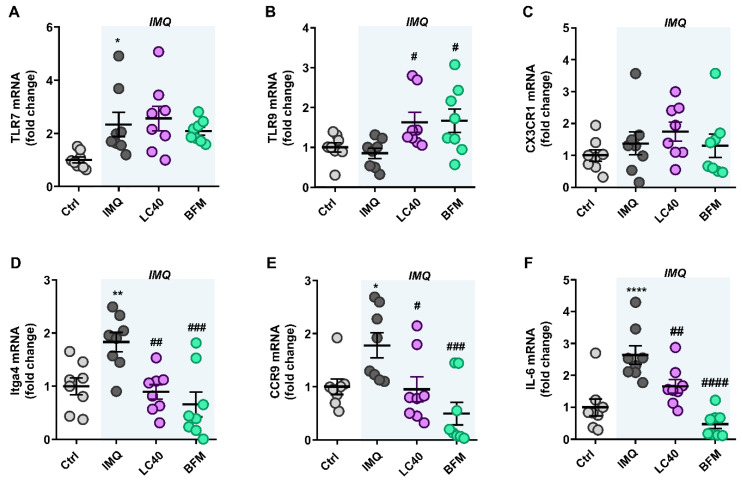
Effects of probiotic treatments on TLR expression and T cells activation in mesenteric lymph nodes (MLNs) from imiquimod-treated mice. mRNA levels of TLR 7 (**A**), TLR9 (**B**), CX3CR1 (**C**), Itga4 (**D**), CCR9 (**E**), and interleukin (IL)-6 (**F**) in MLNs from all experimental groups. Groups: control (Ctrl), Imiquimod (IMQ), IMQ treated with *Lactobacillus fermentum* CECT5716 (LC40), and IMQ-treated with *Bifidobacterium breve* CECT7263 (BFM). Results are expressed as mean ± SEM. *p < 0.05, ** *p* < 0.01, and **** *p* < 0.0001 compared with the Ctrl group. ^#^ *p* < 0.05, ^##^ *p* < 0.01, ^###^ *p* < 0.001, and ^####^ *p* < 0.0001 compared with the IMQ group.

**Figure 5:**
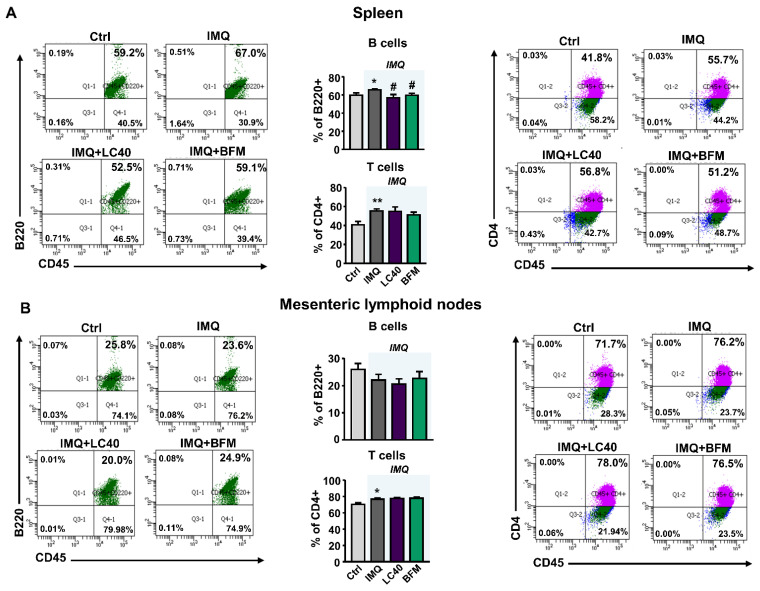
Effects of probiotic treatments on percentage of B and T cells in spleen and mesenteric lymph nodes from imiquimod-treated mice. Total B (B220+), and T lymphocytes in spleen (**A**) and mesenteric lymphoid nodes (**B**) from all experimental groups. Groups: control (Ctrl), Imiquimod (IMQ), IMQ treated with *Lactobacillus fermentum* CECT5716 (LC40), and IMQ-treated with *Bifidobacterium breve* CECT7263 (BFM). Results are expressed as mean ± SEM. * *p* < 0.05 and ** *p* < 0.01 compared with the Ctrl group. ^#^ *p* < 0.05 compared with the IMQ group.

**Figure 6:**
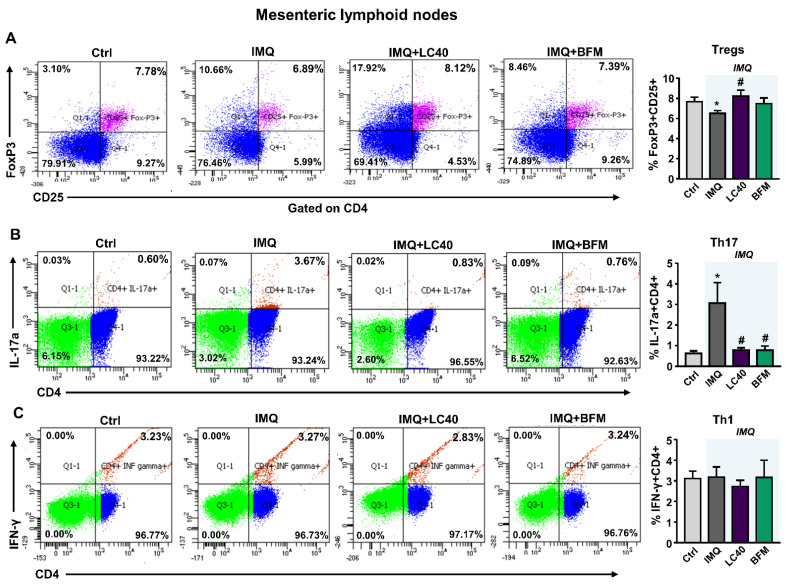
Effects of probiotic treatments on T-cell polarization in mesenteric lymph nodes from imiquimod-treated mice. (**A**) Regulatory T cells (Treg; CD4+ FoxP3+), (**B**) Th17 (CD4+ IL17a+), and (**C**) Th1 (CD4+ interferon-γ+ [IFN-γ+]) cells measured in mesenteric lymphoid nodes from all experimental groups. Groups: control (Ctrl), Imiquimod (IMQ), IMQ treated with *Lactobacillus fermentum* CECT5716 (LC40), and IMQ-treated with *Bifidobacterium breve* CECT7263 (BFM). Results are expressed as mean ± SEM. * *p* < 0.05 compared with the Ctrl group. ^#^ *p* < 0.05 compared with the IMQ group.

TLR-7 activation is linked to an imbalance in T cell subpopulations and high B cells [17]. We determined the mRNA levels of TLR-7 and TLR-9 in MLNs, and found higher TLR7 expression in all groups treated with IMQ as compared with control mice (Figure 4A). By contrast, TLR9 mRNA levels were similar between control and IMQ groups, but were increased by both LC40 and BFM (Figure 4B). Under altered gut mucosal integrity, bacteria can translocate through the intestinal epithelium, which may lead to CX3CR1^+^ cells, such as macrophages and dendritic cells, activation and migration to draining lymph nodes of the lower intestinal tract [33]. Nevertheless, no changes in CX3CR1 mRNA levels were found in MLNs from all groups (Figure 4C). They as well present soluble antigens to naïve CD4+ T cells, lending to T cell activation. Upon activation, these T cells upregulate integrin α4β7 and chemokine receptor CCR9 [34]. We observed increased Itga4 (Figure 4D) and CCR9 (Figure 4E) mRNA levels in the IMQ group, showing T cells activation, which were reduced by both probiotic treatments. Amongst the pro-inflammatory cytokines synthetized by activated T cells, IL-6 is known to boost Th17 proliferation and Treg suppression [35]. We determined the transcriptional levels of IL-6 in MLNs (Figure 4F) and found that they were significantly increased in the IMQ group and reduced by both BFM and LC40. To assess the immunomodulatory effects of the probiotics, we determined B and T cell levels in MLNs and spleen. The relative populations of T cells were higher in both organs from IMQ mice than in organs from control group (Figure 5A,B). Splenic B cells levels were significantly decreased with probiotics in IMQ group (Figure 5A). As expected, the percentages of T-helper (Th) 17 cells (CD4+/IL-17a+) were increased in both organs from IMQ mice (Figure 6 and Figure S3), while Th1 (CD4+/IFN-γ+) cells were also increased in spleens from IMQ group (Figure S3). On the other hand, IMQ treatment led to a reduction in the percentage of T regulatory (Treg, CD25+/FoxP3+) cells in MLNs (Figure 6) and spleens (Figure S3). Again, both probiotics reduced Th17 in MLNs, though, only in the LC40-treated group Treg were elevated (Figure 6). No significant effects of both probiotics were found in the population of Treg, Th17 and Th1 in spleen (Figure S3).

### 3.3. Probiotics Prevent Endothelial Dysfunction, Oxidative Stress and Hypertension

**Figure 7:**
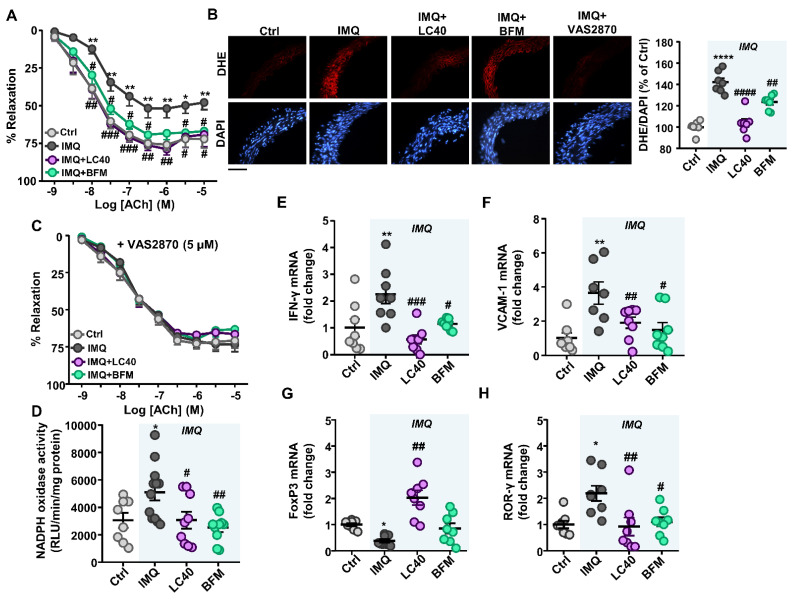
Effects of probiotic treatments on endothelial function, oxidative stress-inflammation and T cells infiltration in aorta from imiquimod-treated mice. Vascular relaxant responses triggered by acetylcholine (ACh), in endothelium-intact aortae pre-contracted by U46619 (10 nM) in the absence (**A**) and in the presence of the specific NADPH oxidase inhibitor VAS2870 (5 μM) (**C**). Top pictures show arteries incubated with dihydroethidium (DHE), which produces a red fluorescence when oxidized to ethidium by ROS. Bottom pictures show blue fluorescence of the nuclear stain 4,6-diamidino-2-phenylindole dichlorohydrate (DAPI; ×400 magnification). Averaged values, mean ± SEM (*n* = 6–9 vessel segments from different mice) of the red ethidium fluorescence normalized to the blue DAPI fluorescence (**B**). NADPH oxidase activity measured by lucigenin-enhanced chemiluminescence (**D**). Aortic expression of pro-inflammatory cytokine, interferon-gamma (IFN-γ) (**E**), vascular cell adhesion molecule-1 (VCAM-1) (**F**), forkhead box P3 (FoxP3) (**G**), and retinoic acid receptor-related orphan receptor (RORγ) (**H**) at the level of mRNA by RT-PCR. Groups: control (Ctrl), Imiquimod (IMQ), IMQ treated with *Lactobacillus fermentum* CECT5716 (LC40), and IMQ-treated with *Bifidobacterium breve* CECT7263 (BFM). Results are expressed as mean ± SEM. * *p* < 0.05, ** *p* < 0.01, and **** *p* < 0.0001 compared with the Ctrl group. ^#^ *p* < 0.05, ^##^ *p* < 0.01, ^###^ *p* < 0.001, and ^####^ *p* < 0.0001 compared with the IMQ group.

The treatment of IMQ mice with both probiotics displayed a rise in the acetylcholine-induced vasorelaxation in comparison with IMQ-treated group (Figure 7A). For all groups, the acetylcholine-induced relaxation was completely inhibited by the eNOS inhibitor L-NAME (Figure S4A), showing that this vessel relaxation triggered by acetylcholine was fully dependent on NO derived from endothelium. In addition, the endothelium-independent vasodilator responses to nitroprusside, which activates the soluble guanylyl cyclase in vascular smooth muscle, presented no significative difference among groups (Figure S4B), indicating no change in vascular smooth muscle NO signalling. Moreover, no significant fluctuations in eNOS gene expression in aorta from all groups were observed (Figure S4C). 

Since ROS synthesis by the NADPH oxidase is a key element in endothelial dysfunction in IMQ-treated mice [17], both ethidium red fluorescence and NADPH oxidase activity were analyzed in aorta from all experimental groups. Positive red nuclei were observed in adventitial, medial and endothelial cells from in aortic rings stained with DHE (Figure 7B). Aortic segments from the IMQ group displayed a clear staining in adventitial, medial and endothelial cells, which was greater than in the control group, which was almost suppressed by the selective NADPH oxidase inhibitor VAS2870. These effects were prevented by LC40 and BFM treatments (Figure 7B). On the other hand, we tested endothelium-dependent relaxation to acetylcholine in the presence of VAS2870. No significant differences among groups were detected after incubation with VAS2870, showing the critical role of increased NADPH oxidase in the endothelial dysfunction found in aorta from IMQ-treated mice (Figure 7C). In addition, NADPH oxidase activity was higher in aortic rings from lupus mice when compared with control mice. Chronic administration of both probiotics reduced significantly the increased NADPH oxidase activity in IMQ mice (Figure 7D).

To determine whether proinflammatory cytokines play a crucial part in the pathogenesis of endothelial dysfunction in SLE, we measured the mRNA level of proinflammatory cytokines and vascular adhesion molecules in aorta from all experimental groups. Again, both probiotics reduced the expression on IFN-γ (cytokines released by Th1) (Figure 7E) and the vascular cell adhesion molecule-1 (VCAM-1) (Figure 7F), which were found elevated in the IMQ model. In addition, we also analyzed the transcript levels of transcription factors, such as retinoic acid receptor-related orphan receptor (ROR)γ and forkhead box P3 (FoxP3), which are involved in the maturation of Th17 and regulatory T cell (Tregs) populations, respectively. The expression levels of these transcription factors in lymphocytes were used as indirect markers of Treg and Th17 cells. FoxP3 and RORγ mRNA levels were decreased and elevated, respectively, in IMQ group, and LC40 administration increased the relative populations of Treg found in aorta from IMQ group (Figure 7G), whereas both LC40 and BFM reduced Th17 infiltration in aorta (Figure 7H). 

**Figure 8:**
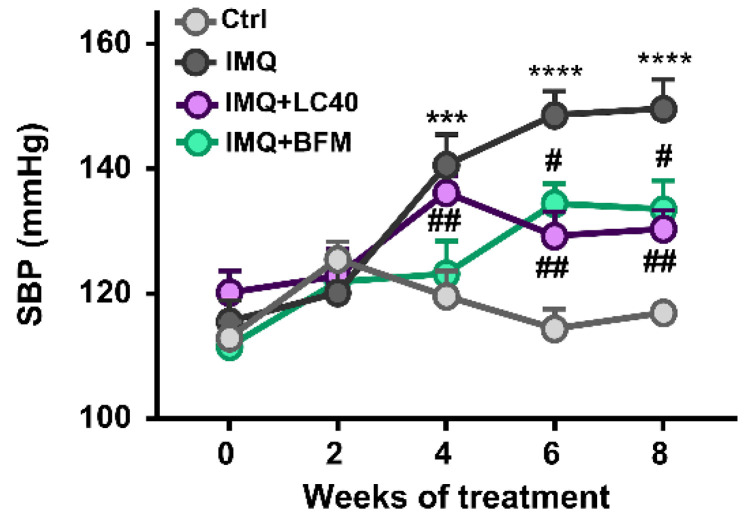
Effects of probiotic treatments on blood pressure in imiquimod-treated mice. Systolic blood pressure (SBP) was determined by tail-cuff plethysmography. Groups: control (Ctrl), Imiquimod (IMQ), IMQ treated with *Lactobacillus fermentum* CECT5716 (LC40), and IMQ-treated with *Bifidobacterium breve* CECT7263 (BFM). Results are expressed as mean ± SEM. *** *p* < 0.011, and **** *p* < 0.0001 compared with the Ctrl group. ^#^ *p* < 0.05, and ^##^ *p* < 0.01 compared with the IMQ group.

Finally, analysing the evolution of SBP, we observed the already stablished hypertension in the IMQ group, which was prevented by both probiotics (Figure 8). Anatomical analysis revealed that a left ventricular weight/tibia length and kidney weight/tibia length index was increased in IMQ-treated mice than in the control group. Interestingly, both probiotics prevented cardiac and renal hypertrophy found in IMQ mice (Table 3).

## 4. Discussion

Our results demonstrate for the first time that the chronic treatment of the probiotics LC40 or BFM prevented hypertension and endothelial dysfunction in a mouse lupus model induced by TLR-7 activation. These findings identify an alternative for the prevention of vascular disorders in SLE based in gut microbiota manipulation using probiotics. The protective effects are the following: (a) a markedly mitigated lupus disease progression as shown by B cells accumulation in spleen, and decreased plasma anti-dsDNA; (b) a restoration the Th17/Treg balance in MLNs; (c) an endothelial function restoration associated to lower vascular Th1, and Th17 infiltration, and vascular oxidative stress; and (d) a significant reduced SBP. 

Our previous works showed that the oral administration of probiotics ameliorates blood pressure in tacrolimus-induced hypertension [36], in spontaneously hypertensive rats [37,38], in mineralocorticoid-induced hypertensive rats [39], and in NZBWF1 mice [19,40]. These data suggest that the effect of probiotics on blood pressure should be closely linked to the composition of intestinal flora. In our experiment, LC40 or BFM administered to IMQ mice prevented the raise of blood pressure, suggesting that changes in gut microbiota might influence the mechanisms involved in the development of hypertension induced after TLR7 activation.

The major changes in gut microbiota found in IMQ-treated mice as compared to their healthy counterparts are an induced intestinal dysbiosis characterized by both a tendence to reduce the F/B ratio and a significant lower α-diversity, measured by numbers of species. Our results are in agreement with the microbiota alterations found in the others animal model of SLE [23], and even to that previously described after administration of the agonist of TLR-7, IMQ [22]. The microbiota in SLE is characterized by an expansion of family *Coriobacteriaceae,* and *Rikenallecea,* and lower populations of *Clostridaceae*. At the genus level, spotlight the change in *Prevotella* and *Desulfovibrio* which were found elevated in SLE model and the reduction in the abundance of *Turicibacter*, *Bifidobacterium*, *Coprobacillus* and *Anaerostipes*. Finally, an increase in the specie *Lactobacillus reuteri*, in faeces from the IMQ group was found, which was also detected translocated in MLN, spleen and liver, linking this phenomenon to the evolution of the pathology. However, no significant changes among groups were found in the abundance of *L. reuteri* (not shown) in our experiment, suggesting no involvement of this bacterium in the development of autoimmunity. This important difference would be related to the differences between mouse strains used by Zegarra-Ruiz et al. (C57Bl/6 mice) [22] and in the present experiment (BALB/c mice). In our experimental conditions, principal component analysis (PCA) of the bacterial taxa in faecal samples displayed a perfect clustering among groups (Ctrl and IMQ). The clusters corresponding to LC40 and BFM were more similar to IMQ. Reduced SCFAs-producing bacteria (acetate and butyrate) seems to be involved in the raise of blood pressure found in spontaneously hypertensive rats, angiotensin II-infused mice and hypertensive subjects [38,41]. We also analysed the content of SCFAs-producing bacteria, acetate-producing bacteria levels were found elevated but neither of the probiotics restored it, ruling out a possible role of SCFAs in the protective effects of these probiotics. BFM and LC40 were detected present in the gut microbiota of animals treated, respectively, and could be responsible of the release of bacterial products, other than SCFAs, involved in blood pressure control.

In addition to these changes, in the microbiota from patients and animal models of SLE takes place a pathophysiological process in the epithelium of the intestinal barrier characterized by impairment in junction proteins like occludin, ZO-1 and claudin, and a higher intestinal permeability, as determined by fluorescein isothiocyanate (known as FITC-dextran) [42]. In fact, our experiment exhibited that the intestinal epithelium is altered in IMQ mice characterized by a low expression of tight junction proteins, which could facilitate the translocation of LPS to the systemic circulation. In addition, the relative abundance of LPS-containing Gram-negative bacteria (Proteobacteria) tended to be higher in the IMQ group as compared to control mice, which could contribute to increase plasma LPS level found in this group. Interestingly, LC40 or BFM treatments can restore the mucosal barrier integrity by increasing the expression of occludin and mucin proteins which function primarily to protect the intestinal epithelium [43]. In contrast with previous data in *lpr* mice and NZBWF1 *Lactobacillus*-treated mice [19,42], we found that both probiotics did not significantly reduce the LPS plasma levels. This data could be related to the absence of change in the relative abundance of LPS-containing Gram-negative bacteria induced by both probiotics. Together, these results indicate that probiotics could improve intestinal mucosal barrier function that is severely altered in lupus mice.

Current evidence suggests that immune system activation is central to the pathogenesis of SLE immune cells, which can affect vascular reactivity, renal function, and blood pressure regulation [44]. In fact, mouse anti-CD20 antibodies (the equivalent of rituximab in humans) have been shown to deplete B cells, distinctly reducing autoantibody levels and prevents the development of hypertension in female NZBWF1 mice, showing the key role of autoantibodies in the control of blood pressure in SLE [44]. TLR-7 activation increased type I IFN secretion in the blood increasing autoimmunity [15]. We also found higher plasma IFNα levels in the IMQ group, as compared with control, which were unaltered by both probiotic treatments, indicating that changes in anti-dsDNA induced by these probiotics were independent of TLR7-type I IFN pathway. However, we found an increased amount of B cells in spleen in IMQ mice than in the control group, which was counteracted after LC40 and BFM treatment in lupus mice, leading to reduced plasma anti-dsDNA levels. Additionally, an imbalance between anti-inflammatory Treg and inflammatory Th17 cells is widely recognized as being causative in the establishment of both human SLE and murine lupus [23]. Our data are in agreement with a previous study by Robles-Vera et al. [17], that points to the ability of IMQ to promote T-cell proliferation towards Th17 cells in spleens, together with a reduction in Treg cell number, provides an additional mechanism that may take part in the hypertension induced by IMQ, in addition to its direct deleterious effects on blood vessels. In fact, IL17 neutralization in vivo improved endothelial dysfunction and reduced SBP. Furthermore, we discovered that IMQ lowered Treg and augmented Th17 cells in MLNs. Finally, preventing T-cell polarization with LC40 or BFM improved endothelial dysfunction induced by IMQ. 

The probiotic capabilities of commensal bacteria such as lactobacilli and bifidobacteria are likely to be determined at least in part by their effects on dendritic cells. TLRs are pattern-recognition receptors that identify microbial components and trigger an innate immune response. Several data showed a pathogenic role for TLR7, with an opposing, protective role for TLR9 in the induction of systemic autoimmunity [45]. Recent studies point to the possible role microbial DNA from probiotic microorganisms might play in the protective effects of these agents, and that this protection requires activation of the microbial DNA receptor TLR9 [46,47]. In this experiment, we detected a rise in TLR9 expression in MLNs from IMQ mice treated with both probiotics, which could be involved in the reduced T cells activation and Th17 polarization induced by both LC40 and BFM. In addition, reduced Th17 populations induced by both probiotics could be associated with a rise in IL-18 secretion in colonic tissue, an important cytokine that downregulates colonic Th17 cell differentiation in MLNs [35].

It is becoming more and more evident that hypertension is often linked to impaired endothelial function. We have demonstrated that chronic probiotic administration prevented the decreased responses to acetylcholine shown in aortae from control IMQ mice. Remarkably, the improvement in acetylcholine relaxation with both probiotics in IMQ was depleted by L-NAME, pointing to a protective role in NO bioactivity. In the present study, the relaxant response to the activator of soluble guanylate cyclase SNP was similar in the aorta from all of the experimental groups, indicating that alterations in cGMP pathway do not appear to be involved in endothelial dysfunction in lupus mice. These data are also consistent with our previous studies that showed an impaired aortic endothelium-dependent relaxation response to acetylcholine in NZBWF1 mice [19,25] and in TLR-7 dependent mouse model [17].

An important mechanism in endothelial dysfunction includes the vascular production of ROS, especially O_2_^-^, which rapidly reacts with NO, inactivating it [48]. In our study, aortae from IMQ-treated mice displayed increased ROS levels, both probiotics abolished this increase. The activity of the NADPH oxidase, commonly referenced as the main source of O_2_^−^ in the vascular wall, was evidently high in IMQ mice. ROS production by the NADPH oxidase is a crucial part of endothelial dysfunction in SLE, incubation with the selective NADPH oxidase inhibitor VAS2870 improved the aortic endothelium-dependent relaxation to acetylcholine in IMQ to similar levels found in control mice. Our data suggest that a decrease in O_2_^-^ levels in the vascular wall, and the subsequent prevention of NO inactivation, are part of a crucial pathway in the protective effects of probiotics on endothelial function in SLE disease. High ROS production levels have also been involved in the raise of blood pressure in TLR7-model since chronic treatment with a combination of antioxidants reduced blood pressure [17]. 

TLR-7 activation induced by IMQ enhances vascular inflammation and inflammatory cells infiltration, which leads to vascular dysfunction in SLE, largely driven by immune dysregulation [9,10]. In agreement with this, our data have shown that the mRNA expression of VCAM-1 and the proinflammatory cytokine IFN-γ were increased in aortae from the IMQ group. Additionally, LC40 or BFM, which reduced ROS levels, also prevented the increase in the mRNA levels of IFN-γ and VCAM-1 caused with IMQ. Remarkably, blood pressure was decreased in LC40- or BFM-treated mice as compared with the IMQ group. Furthermore, increased vascular inflammation and oxidative stress, mediated in part by IL-17, as key factors contributing to hypertension in this TLR7-driven lupus autoimmunity model [17]. We found increased Th17 infiltration in aorta from IMQ mice, which was abolished by both probiotics, possibly related to the change in T cells polarization induced by LC40 and BFM in MLNs. Th17 is known to increase the NADPH oxidase activity through the production of IL-17a [49]. Moreover, LC40 also increased Treg accumulation in the aorta. It is well stablished that IL-10 released by Treg improves endothelial dysfunction through inhibition of vascular NADPH oxidase activity improving vascular oxidative stress [50], which contributes to reduce blood pressure in IMQ-treated mice. Overall, our results suggested that probiotics reduced Th17 and increased Treg aortic infiltration, which resulted in lower NADPH oxidase driven-ROS production, increasing NO bioavailability and, consequently, reducing endothelial dysfunction and the rise in blood pressure. This experiment supports the hypothesis that modifications in the gut microbiota, which in turn reduced immune activation, may have a crucial part in the observed effects of LC40 or BFM in IMQ-treated mice.

In conclusion, we have found that probiotics LC40 and BFM prevented the development of hypertension and endothelial dysfunction in IMQ mice through a decrease in the vascular oxidative stress. These protective effects may be attributable to a reduction of SLE activity and vascular inflammation, possibly due to reduced Th17 and high Treg populations in MLNs, restoring Th17/Treg balance in vascular tissues.

## Figures and Tables

**Table 1:** Oligonucleotides for real-time RT-PCR.

mRNA Targets	Descriptions	Sense	Antisense
*eNOS*	endothelial nitric oxide synthase	ATGGATGAGCCAACTCAAGG	TGTCGTGTAATCGGTCTTGC
*IFN-γ*	Interferon-gamma	GCCCTCTCTGGCTGTTACTG	CCAAGAGGAGGCTCTTTCCT
*TNF-α*	Tumor necrosis factor-alpha	CTACTCCCAGGTTCTCTTCAA	GCAGAGAGGAGGTTGACTTTC
*Muc-2*	Mucin-2	GATAGGTGGCAGACAGGAGA	GCTGACGAGTGGTTGGTGAATG
*Muc-3*	Mucin-3	CGTGGTCAACTGCGAGAATGG	CGGCTCTATCTCTACGCTCTCC
*Occludin*	Occludin	ACGGACCCTGACCACTATGA	TGGAGATGAGGCTTCTGCTT
*ZO-1*	Zonula occludens-1	GGGGCCTACACTGATCAAGA	TGGAGATGAGGCTTCTGCTT
*IL-18*	Interleukin-18	GACTCTTGCGTCAACTTCAAGG	CATGGACCGCTTCCCATA
*IL-6*	Interleukin-6	ACTTCACAAGTCCGGAGAGG	TTTCTGCAAGTGCATCATCG
*VCAM-1*	Vascular cell adhesion molecule-1	CTTCCAGAACCCTTCTCAG	GGGACCATTCCAGTCACACTT
*FoxP3*	Forkhead box P3	AGGCACTTCTCCAGGACAGA	CTGGACACCCATTCCAGACT
*ROR-γ*	Retinoid-related orphan receptor-gamma	GCCTACAATGCCAACAACCACACA	TGATGAGAACCAAGGCCGTGTAGA
*TLR-7*	Toll-like receptor-7	GCCATCCAGCTTACATCTTCT	TTTGACCCAGGTAGAGTGTTTC
*TLR-9*	Toll-like receptor-9	CTACAACAGCCAGCCCTTTA	GGACACACGGGTATGAATGT
*CX3CR-1*	CX3C chemokine receptor 1	TGAGTGACTGGCACTTCCTG	CGAGGACCACCAACAGATTT
*CCR9*	C-C chemokine receptor type 9	CCAGGAAATCTCTGGTCTGC	CTGTGGAAGCAGTGGAGTCA
*Itga4*	Integrin alpha-4	TGTGCAAATGTACACTCTCTTCCA	CTCCCTCAAGATGATAAGTTGTTCAA
*RPL13*	Ribosomal protein L13	CCTGCTGCTCTCAAGGTTGTT	TGGTTGTCACTGCCTGGTACTT
*GAPDH*	Glyceraldehyde-3-Phosphate Dehydrogenase	ACCACAGTCCATGCCATCAC	TCCACCACCCTGTTGCTGTA

**Table 2:** Effects of probiotic treatments on phyla proportion (% of total reading).

Phylum	Ctrl (*n* = 8)	IMQ (*n* = 8)	IMQ + LC40 (*n* = 8)	IMQ + BFM (*n* = 8)
Tenericutes	1.5 ± 0.2	0.3 ± 0.1	0.4 ± 0.2	0.9 ± 0.4
Cyanobacteria	0.5 ± 0.1	1.0 ± 0.1	0.9 ± 0.1	0.7 ± 0.1
Proteobacteria	3.6 ± 0.3	4.4 ± 0.3	4.6 ± 0.8	3.7 ± 0.3
Bacteroidetes	49.9 ± 4.3	69.0 ± 3.9 ****	63.0 ± 7.4	66.0 ± 5.9
Firmicutes	44.0 ± 4.5	24.9 ± 3.9 ****	30.4 ± 6.8	28.0 ± 5.5
Others	0.6 ± 0.0	0.5 ± 0.1	0.8 ± 0.2	0.7 ± 0.1

Results are shown as mean ± SEM. All parameters were determined in control mice (Ctrl) and in imiquimod (IMQ) treated with vehicle, *Lactobacillus fermentum* CECT5716 (LC40), or *Bifidobacterium breve* CECT7263 (BFM). **** *p* < 0.0001 vs. Ctrl group.

**Table 3:** Morphological parameters of all experimental groups.

Variables	Ctrl (*n* = 8)	IMQ (*n* = 12)	IMQ + LC40 (*n* = 9)	IMQ + BFM (*n* = 10)
BW (g)	21.4 ± 0.4	20.8 ± 0.6	19.5 ± 2.3	21.1 ± 0.7
HW/TL (mg/cm)	5.28 ± 0.16	5.58 ± 0.15	5.37 ± 0.14	5.43 ± 0.09
LVW/TL (mg/cm)	3.49 ± 0.12	3.87 ± 0.12 *	3.44 ± 0.12 ^#^	3.49 ± 0.9 ^#^
KW/TL (mg/cm)	6.10 ± 0.19	7.50 ± 0.18 **	6.98 ± 0.10 ^#^	6.79 ± 0.21 ^#^
LW/TL (mg/cm)	53.77 ± 2.4	61.96 ± 4.12	67.58 ± 4.4	64.59 ± 3.7
SW/TL (mg/cm)	4.37 ± 0.43	27.97 ± 1.86 **	25.84 ± 1.09	19.19 ± 1.64 ^##^
CW/CL (mg/cm)	21.53 ± 1.07	16.94 ± 0.64 **	17.34 ± 0.81	17.14 ± 0.82

BW, Body weight; HW, Heart weight; KW, Kidney weight; LVW, Left ventricular weight; LW, Liver weight; SW, Spleen weight; TL, Tibia length; CW, Colon weight; CL, Colon length. Results are expressed as mean ± SEM. All parameters were assessed in control mice (Ctrl) and in imiquimod (IMQ) treated with vehicle, *Lactobacillus fermentum* CECT5716 (LC40), or *Bifidobacterium breve* CECT7263 (BFM). * *p* < 0.05 and ** *p* < 0.01 vs. Ctrl group. ^#^ *p* < 0.05 and ^##^ *p* < 0.01 vs. IMQ group.

## Supplementary Materials

The following are available online at https://www.mdpi.com/article/10.3390/nu13082669/s1, Figure S1: Schema for flow cytometric identification of B, T, T regs, Th17, and Th1 cells in mesenteric lymph nodes and spleen, Figure S2: Comparisons of microbiome changes in control (Ctrl) versus Imiquimod (IMQ) mice, Figure S3: Effects of probiotic treatments on T-cell polarization in spleens from imiquimod-treated mice, Figure S4: Effects of probiotic treatments on vascular nitric oxide pathway from imiquimod-treated mice.

## Abbreviations

Ach, acetylcholine; BFM, *Bifidobacterium breve* CECT7263; CFU, colony-forming units; Ctrl, control; DAPI, 4,6-diamidino-2-phenylindoledichlorohydrate; DHE, dihydroethidium; eNOS, endothelial nitric oxide synthase; F/B, *Firmicutes/Bacteroidetes*; FoxP3, forkhead box P3; GAPDH, glyceraldehyde-3-phosphate dehydrogenase; IFN, interferon; IMQ, imiquimod; LAL, limulus amebocyte lyste; LC40, Lactobacillus *fermentum* CECT5716; LDA, linear discriminant analysis; L-NAME, N^G^-nitro-L-arginine methyl ester; LPS, lipopolysaccharide; MLN, mesenteric lymph nodes; MUC, mucin; NIH, National Institutes of Health; NO, nitric oxide; O_2_^−^, superoxide anions; PAMPs, pathogen-associated molecular patterns; PCA, principal component analysis; PLS, partial least square; QIIME, quantitative insights into microbial ecology; RLU, relative luminescence units; ROR, retinoic acid receptor-related orphan receptor; ROS, reactive oxygen species; RPL13a, ribosomal protein L13a; Rpm, revolutions per minute; RT, real-time; SBP, Systolic blood pressure; SCFAs, short chain fatty acids; SLE, Systemic lupus erythematosus; SNP, sodium nitroprusside; Th, T-helper; TLRs, Toll-like receptors; Tregs, regulatory T cell; VCAM-1, vascular cell adhesion molecule-1; ZO-1, zonula occludens-1
