# Supplementary material for: Probiotics Prevent Hypertension in a Murine Model of Systemic Lupus Erythematosus Induced by Toll-Like Receptor 7 Activation
Source: Nutrients. 2021 Jul 31;13(8):2669. doi: 10.3390/nu13082669 (PMC8399640; doi:10.3390/nu13082669)
Supplement: Supplementary file 1 [file nutrients-13-02669-s001.zip › nutrients-1277417-supplementary.pdf]

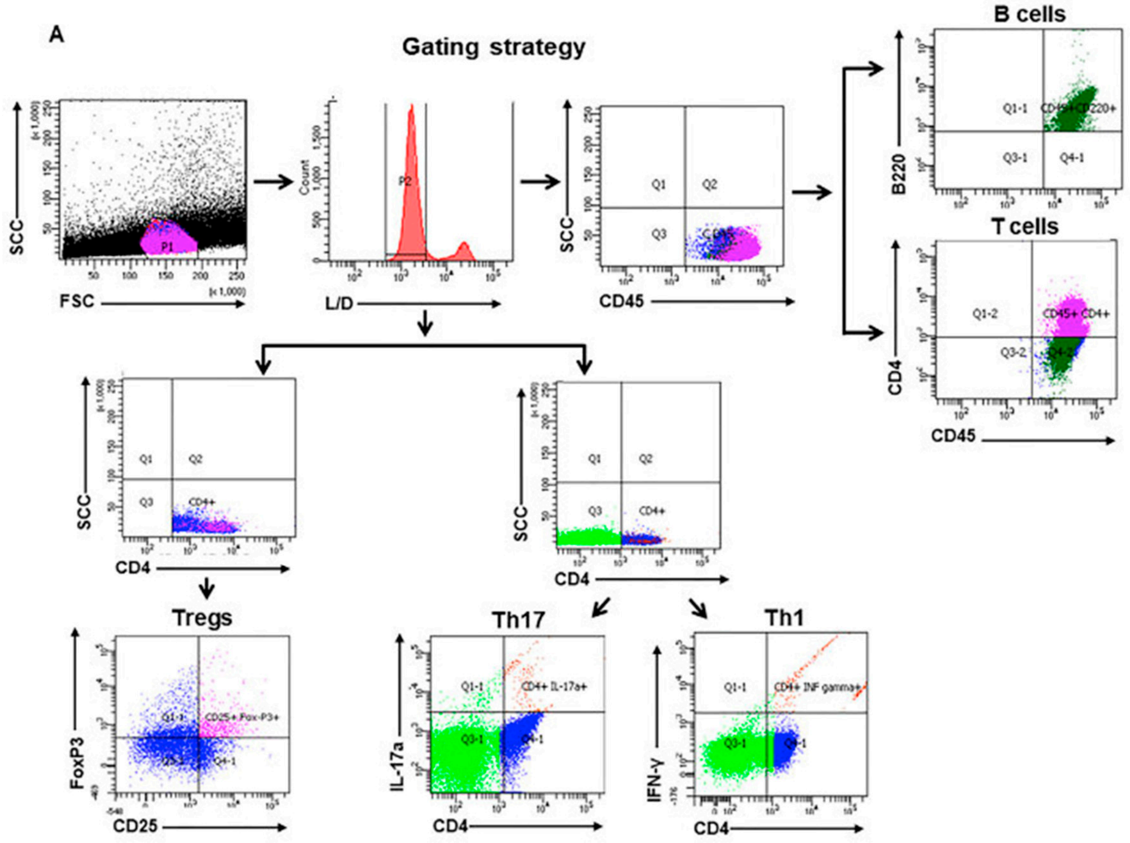

Figure S1. Schema for flow cytometric identification of B, T, T regs, Th17, and Th1 cells in mesenteric lymph nodes and spleen.

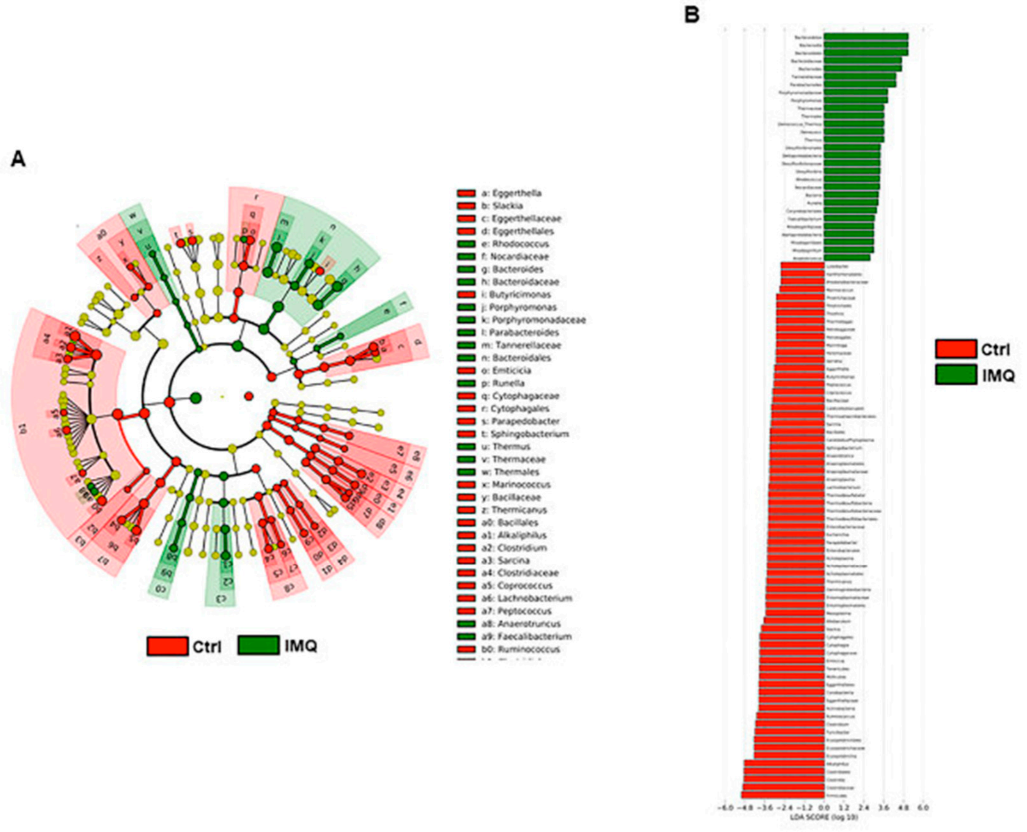

Figure S2. Comparisons of microbiome changes in control (Ctrl) versus Imiquimod (IMQ) mice. (**A**) Cladograms show the significantly enriched taxa, the taxa are identified in the key to the right of each panel. The larger the circles, the greater the difference in abundance between the groups. (**B**) Linear discriminant analysis effects size (LEfSe) identified significantly different bacterial taxa enriched in each cohort at LDA Score > 2, p<0.05 (red bars Ctrl enriched, green bars IMQ enriched). n = 8 mice per treatment group in each comparison.

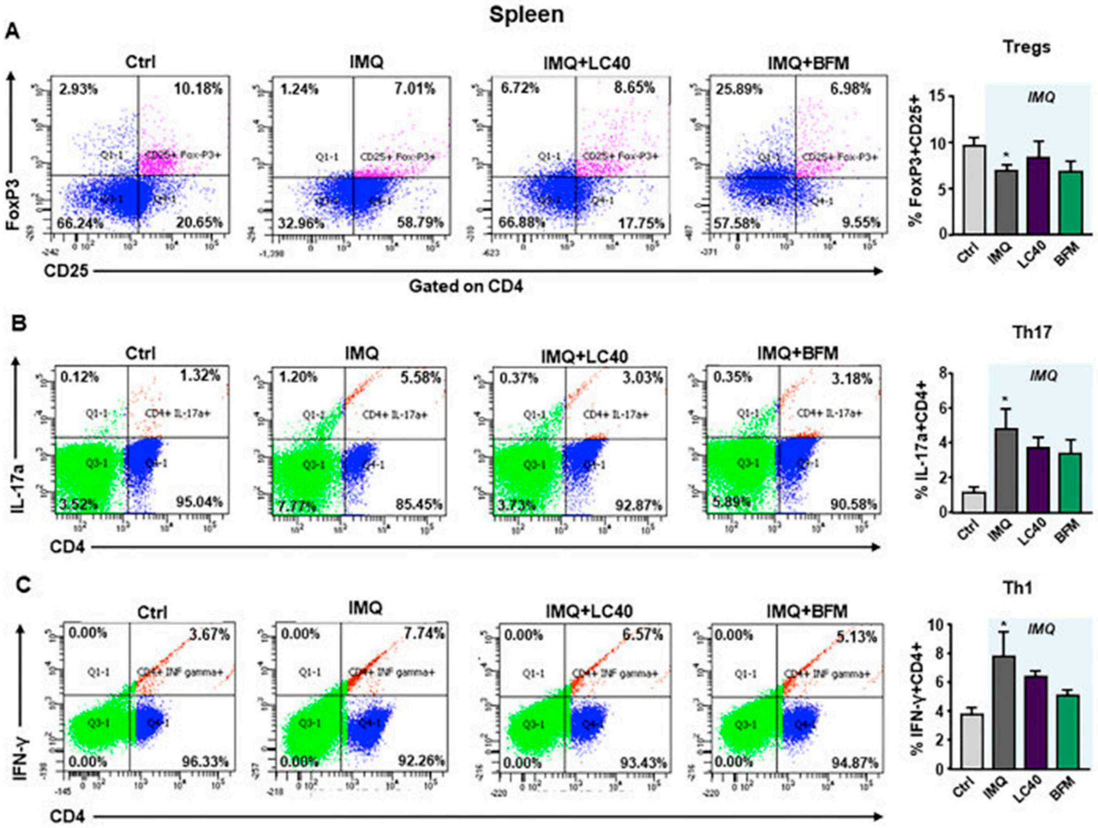

Figure S3. Effects of probiotic treatments on T-cell polarization in spleens from imiquimod-treated mice. (A) Regulatory T cells (Treg; CD4+ FoxP3+), (**B**) Th17 (CD4+ IL17a+), and (**C**), Th1 (CD4+ interferon-γ+ [IFN-γ+]) cells measured in spleens from all experimental groups. Groups: control (Ctrl), Imiquimod (IMQ), IMQ treated with *Lactobacillus fermentum* CECT5716 (LC40), and IMQ-treated with *Bifidobacterium breve* CECT7263 (BFM). Results are expressed as mean ± SEM. *P< 0.05 compared with the Ctrl group.

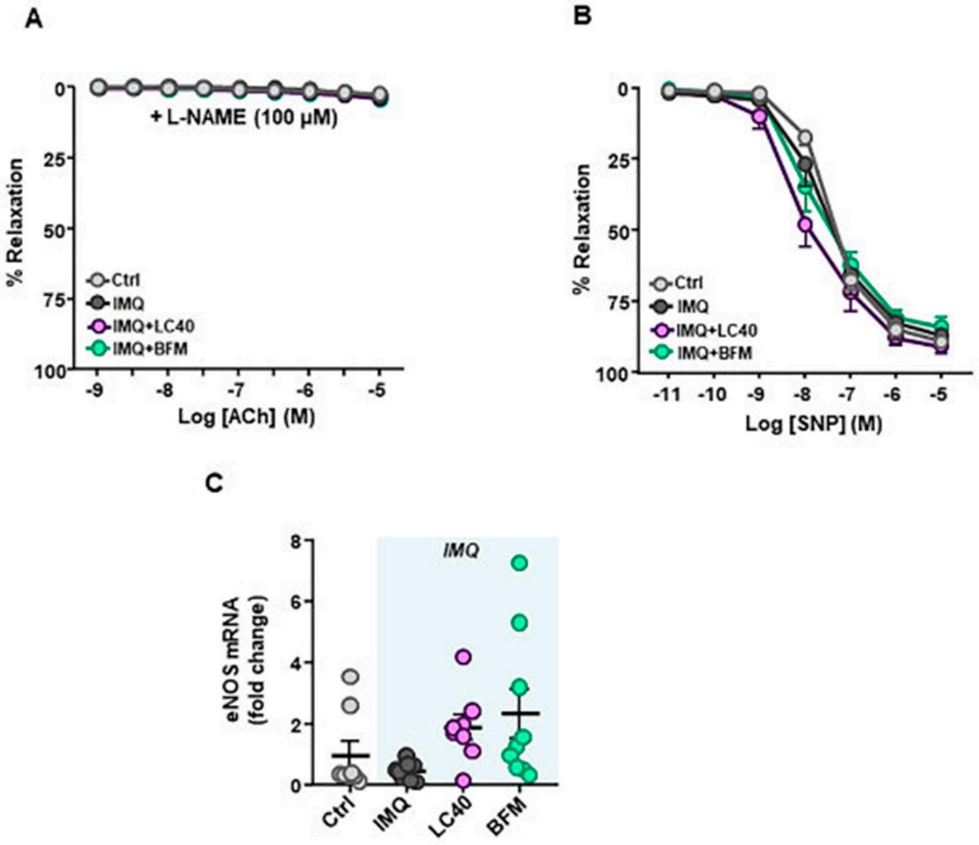

Figure S4. Effects of probiotic treatments on vascular nitric oxide pathway from imiquimod-treated mice. (**A**) Vascular relaxant responses induced by acetylcholine (ACh), in endothelium-intact aortae pre-contracted by U46619 (10 nM) in the presence of N^G-nitro-L-arginine methyl (L-NAME). (**B**) Endothelium-independent vasodilator responses to sodium nitroprusside (SNP) in endothelium-denuded aortae pre-contracted by U46619 (10 nM). **(C)** mRNA levels of endothelial nitric oxide synthase (eNOS) in aorta from all experimental groups. Groups: control (Ctrl), Imiquimod (IMQ), IMQ treated with *Lactobacillus fermentum* CECT5716 (LC40), and IMQ-treated with *Bifidobacterium breve* CECT7263 (BFM). Results are expressed as mean ± SEM.
